# Biochemical Characterization of a Bifunctional Enzyme Constructed by the Fusion of a Glucuronan Lyase and a Chitinase from *Trichoderma* sp.

**DOI:** 10.3390/life10100234

**Published:** 2020-10-08

**Authors:** Zeineb Baklouti, Cédric Delattre, Guillaume Pierre, Christine Gardarin, Slim Abdelkafi, Philippe Michaud, Pascal Dubessay

**Affiliations:** 1 CNRS, SIGMA Clermont, Institut Pascal, Université Clermont-Auvergne, FS-63000 Clermont-Ferrand, France; zeineb.baklouti@etu.uca.fr (Z.B.); cedric.delattre@uca.fr (C.D.); guillaume.pierre@uca.fr (G.P.); christine.gardarin@uca.fr (C.G.); philippe.michaud@uca.fr (P.M.); 2 Département Génie Biologique, Université de Sfax, Unité de Biotechnologie des Algues, Ecole National d’Ingénieurs de Sfax, 3018 Sfax, Tunisia; slim.abdelkafi@enis.tn; 3 Institut Universitaire de France (IUF), 1 rue Descartes, 75005 Paris, France

**Keywords:** chitinase, glucuronan lyase, bifunctional enzyme, enzymatic efficiency, polysaccharide

## Abstract

Bifunctional enzymes created by the fusion of a glucuronan lyase (TrGL) and a chitinase (ThCHIT42) from *Trichoderma* sp. have been constructed with the aim to validate a proof of concept regarding the potential of the chimera lyase/hydrolase by analyzing the functionality and the efficiency of the chimeric constructions compared to parental enzymes. All the chimeric enzymes, including or nor linker (GGGGS), were shown functional with activities equivalent or higher to native enzymes. The velocity of glucuronan lyase was considerably increased for chimeras, and may involved structural modifications at the active site. The fusion has induced a slightly decrease of the thermostability of glucuronan lyase, without modifying its catalytic activity regarding pH variations ranging from 5 to 8. The biochemical properties of chitinase seemed to be more disparate between the different fusion constructions suggesting an impact of the linkers or structural interactions with the linked glucuronan lyase. The chimeric enzymes displayed a decreased stability to temperature and pH variations, compared to parental one. Overall, TrGL-ThCHIT42 offered the better compromise in terms of biochemical stability and enhanced activity, and could be a promising candidate for further experiments in the field of fungi Cell Wall-Degrading Enzymes (CWDEs).

## 1. Introduction

The filamentous fungi belonging to *Trichoderma* genus have been considered with interest for their use as enzymatic producer for biocontrol strategies [1]. The antagonistic actions exerted by *Trichoderma* against fungal phytopathogens involve indirect mechanisms, such as competition for nutrients or space, modification of environmental conditions, induction of plant defensive properties and antibiosis, or directly mechanisms encompassing mycoparasitism [2,3]. The production and secretion by *Trichoderma* of Cell-Wall Degrading Enzymes (CWDEs), among which chitinases [4,5], glucanases [6,7,8] and proteases [9,10,11] are the most representative, playing a major and significant role in targeted host invasion [12,13]. 

**Figure 1:**
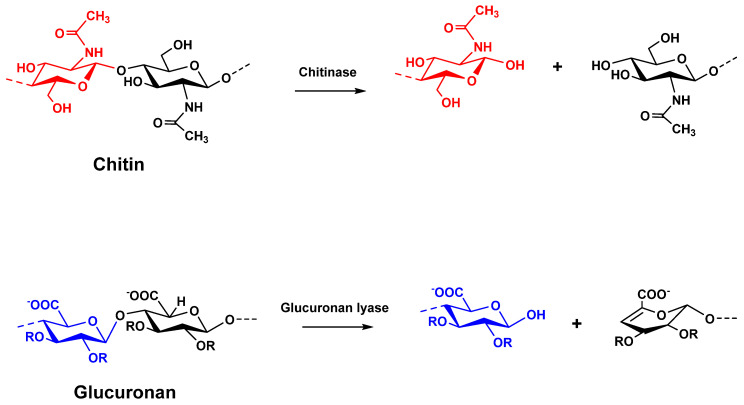
Chitinase and glucuronan lyase mechanism (with R = H and/or acetyl group).

Chitinases are polysaccharide hydrolases categorized into glycoside hydrolase (GH) 18, 19, 20, 23 and 48 families of the CAZy database (http://www.cazy.org/). They catalyze the hydrolysis of β-(1,4) linkages in chitin (Figure 1), an abundant polysaccharide, found as a major structural compound of fungi cell wall [14,15]. Chitins are polymers composed of β(1,4) linked N-acetyl-d-glucosamine (Glc*p*NAc) and glucosamine (Glc*p*N). Native chitins include up to 90% of Glc*p*NAc in their structure. When the degree of acetylation of chitin is lower than 40%, the term chitosan is used. However, authors use rather the “deacetylation degree” to characterize chitosans. Chitinases have been classified in two groups: Endochitinases (E.C.3.2.1.14) which cleave chitin at internal sites forming dimeric or low molecular mass multimers of N-acetyl-glucosamine (GlcNAc) (chitotriose, chitotetraose), and exochitinases comprising both chitobiosidases (E.C.3.2.1.52) catalyzing the release of di-acetylchitobioside from the non-reducing end of chitin, and β-(1,4)-*N*-acetylglucosaminidases (GlcNAcases) (E.C.3.2.1.52) generating monomers of GlcNAc from the products of endochitinase and chitobiosidase [16]. If many GlcNAcases (Nag1, Exc2, Tvnag1 and -2, Chit73, Chit102) identified from *T. harzanium*, *T. atroviride*, *T. asperellum* and *T. reesei* [12,17,18] were known to be functionally active on chitin degradation during mycoparasitism: some of them, such as Nag1 and Chit102, were also involved in the induction of the expression of other chitinolytic enzymes in presence of pathogen [19]. In addition, the over-expression by *Trichoderma* spp. of endochitinases Chit33, Chit36 and Chit42, originally characterized from *T. harzanium* [20], significantly enhanced antagonistic activity of transformants against *Rhizoctonia solani*, *Fusarium oxysporum* and *Sclerotium rolfsii* [9,21,22], with chit42 considered as a key enzyme. 

Among the enzymes produced by *Trichoderma* and secreted in extracellular medium, a glucuronan lyase (E.C.4.2.2.14) was purified and characterized from *Trichoderma* sp. GL2 [23,24]. This lyase cleaves randomly glucuronan polysaccharide, a polymer of β-(1-4)-linked d-glucuronic acids, by a β-elimination mechanism to produce unsaturated oligoglucuronans (Figure 1). Although its role remains unknown, several hypotheses might be considered, notably the contribution of glucuronan lyases in the assimilation of celluloses which are oxidized by lytic polysaccharide monooxygenases (LPMO) produced from bacteria and fungi [25]. Another hypothesis may be to consider this enzyme as a CWDE. Although glucuronan was mainly described as exopolysaccharides in several bacteria [26,27], fragments of glucuronan polysaccharides were also reported as a representative cell-wall component of specific phytopathogens belonging to Mucorales, such as *Mucor* and *Rhizopus* genus [28,29].

Chitinase and glucuronan lyase enzymes could then be attractive as potential biocontrol agent. Several arguments can be advanced for their potential interest in reducing fungi development: i chitin and glucuronan are both components of the cell wall of some phytopathogens (Mucorales), ii chitinase have been demonstrated antagonistic agent against fungi, and iii the anti-fungal properties of glucuronan lyase have never been studied. Moreover, the rise of genetic engineering to produce fusion enzymes led to chimeric enzymes with improved biochemical and catalytic properties [30]. In this study, we have elaborated upon the construction of bi-catalytic chimeric enzymes consisting of the fusion of the glucuronan endolyase from *T. reseei* (TrGL) (E.C.4.2.2.14) and the endochitinase ThCHIT42 from *T. harzanium* (ThCHIT42) (E.C.3.2.1.14), targeting two different polysaccharides of the cell wall of Mucorales fungi. The main goal was to validate a proof of concept regarding the functionality of the chimera lyase/hydrolase on original substrates, and the added value in terms of biochemical and catalytic properties provided by the fusion of two enzymes, without synergistic activities but acting onto independent substrates. 

## 2. Materials and Methods 

### 2.1. Plasmids Constructions and Pichia Pastoris Transformation

Plasmid constructions related to the expression of single protein (TrGL or CHIT42) and fusion proteins (TrGL-CHIT42) were carried out in pPICZαA plasmid (Invitrogen®). Three chimeric TrGL-ThCHIT42 constructions were carried out. The first one, called pPICZαA/*TrGL- Thchit42*, consisted of TrGL directly fused with the N terminus of ThCHIT42. The two others had a linker sequence inserted between the enzyme coding DNA sequences, (GGGGS)_1_ for pPICZαA/*TrGL-sp1-Thchit42* and (GGGGS)_2_ for pPICZαA/*TrGL-sp2-Thchit42*. The genes encoding TrGL (GenBank: AB440264.1) and ThCHIT42 (GenBank: AAA18167.1) were, respectively, obtained by PCR amplification, using Pfu DNA polymerase (Promega^TM^), from *Trichoderma reseei* genomic DNA and Puc57Opt-*ThChit42* plasmid, containing codon optimized *Chit42* gene for *Pichia pastoris* expression (synthesized by Proteogenix Inc., France). For both TrGL and ThCHIT42, native peptide signal related to secretion was removed. First, the 717 bp coding region of TrGL was PCR-amplified with specific primers XTrGL-Fwd and XbTrGL-Rev, containing, respectively, the XhoI and XbaI restriction sites for the insertion in pPICZαA leading to pPICZαA/*TrGL* plasmid (Table 1). The pPICZαA/*ThChit42* plasmid was obtained with the insertion in EcoRI-XbaI sites of pPICZαA, of a 1169 bp fragment encoding CHIT42, amplified with EThchit42-Fwd and XbThchit42-Rev primers (Table 1). For the fusion of glucuronan lyase-chitinase enzymes (TrGL-ThCHIT42), three different plasmids were constructed by sub-cloning, into XbaI site of pPICZαA/TrGL, the *Thchit42* gene amplified by PCR using the following primers (Table 1): Xbchit42-Fwd and Xbchit42-Rev leading to pPICZαA/*TrGL-Thchit42* plasmid; Xbsp1chit42-Fwd and Xbchit42-Rev resulting in pPICZαA/*TrGL-sp1-Thchit42* plasmid; Xbsp2chit42-Fwd and Xbchit42-Rev generating pPICZαA/*TrGL-sp2-Thchit42* plasmid. Sp1 and sp2 sequences are related to linker sequences encoding, respectively, for (GGGGS)_1_ and (GGGGS)_2_ amino acids motif, which were introduced to reduce potential steric hindrance that may limit the folding functionally of fused enzymes. For all fusion constructions, the bacterial clones with the *Thchit42* gene inserted in correct orientation were selected by PCR and confirmed by DNA sequencing.

Plasmid constructions, linearized with SacI, were transformed by chemical method in *P. pastoris* (strain KMH71) using EasySelect^TM^ Pichia expression Kit (Invitrogen®) according to manual recommendations. Recombinant clones were selected on YPDS (Yeast Extract Peptone Dextrose)-agar plates supplemented with zeocin (250 µg/mL). Several clones were screened for the expression of recombinant proteins by detecting glucuronan lyase and/or chitinase activities in culture supernatant from BMMY (Buffered Methanol-complex Medium) culture induced with methanol (0.5%) during 48 h. 

### 2.2. Production and Purification of Recombinant Parental and Chimeric Enzymes

Enzyme productions were carried out from one selected recombinant clone expressing parental or chimeric enzymes. Recombinant *P. pastoris* were cultivated at 30 °C in 400 mL of BMGY (Buffered Glycerol-complex Medium) during 16 h until reaching ~4 A_600_, concentrated in 100 mL of BMMY (0.5% methanol) and then cultivated for 72 h with regular methanol addition every 24 h. Culture supernatants were collected by centrifugation (3000 g for 15 min) and recombinant enzymes in supernatant were concentrated to 20 mL on 3 kDa ultrafiltration membrane using Stirred Ultrafiltration Cell (Millipore®). Parental and chimeric His-tagged enzymes were then purified by Ion Metal Chromatography Affinity (IMAC) (Protino® Ni-NTA, Macherey-Nagel®), and analyzed by SDS-PAGE (12%, wt/vol.) for the validation of proteins integrity and purity. Protein concentrations were determined by Bradford method using bovine serum albumin as standard.

### 2.3. Enzyme Assays 

The glucuronan lyase activity was spectrophotometrically quantified measuring for 5 min the increase of A_235_ related to unsaturated products. Unless otherwise specified, standard reactions were performed at 25 °C in 50 mM potassium acetate buffer (pH 5.5) with solubilized deacetylated glucuronan substrate (2 g/L). Hydrolytic velocity was monitored for different substrate concentrations (2 g/L, 0.5 g/L, 0.25 g/L and 0.125 g/L). One unit of glucuronan lyase activity (U) was defined as that corresponding to the production of 1 µmol of unsaturated products per minute. The molar extinction coefficient of ∆-(4,5)-unsaturated oligoglucuronan (Degree of Polymerization of 3) was assumed to be 4931 M^−1^ cm^−1^ [23]. The chitinolytic activity was evaluated using Chitinase Assay Kit (from SIGMA-Aldrich) providing three different substrates suitable for the quantification of exo and endo-chitinase activities (4-Nitrophenyl N,N’-diacetyl-β-d-chitobioside and 4-Nitrophenyl N-acetyl-β-d-glucosaminide for exo-activity, and 4-Nitrophenyl β-d-N,N’,N’’-triacetylchitotriose for endo-activity). The reaction was performed at 37 °C for 30 min, in a reaction mixture (final volume 200 µL) composed of substrate solution (0.5 mg/mL, pH 4.8) and appropriate volume of enzyme. The chitinase activity was estimated measuring A_405_ (release of p-nitrophenol from substrate hydrolysis). One unit of enzyme was defined as the formation of 1 µmol of p-nitrophenol per min at 37 °C.

Optimal temperatures of enzyme activity for parental and chimeric enzymes were analyzed according to standard conditions for glucuronan lyase activity. A range of temperature from 20 to 65 °C was tested using parental and chimeric enzymes respecting equimolarity (0.108 mM final). For chitinase activity, a colloidal chitin solution (2 g/L, potassium phosphate 70 mM, pH 6) was used for determining the optimal temperature. The colloidal chitin was prepared from shrimp chitin (SIGMA, degree of acetylation 95%) as described by Kidibule et al. [31]. Enzymes were incubated at the final concentration of 0.108 mM, in colloidal chitin solution under agitation (Radley system, 236 rpm) during 2 h at different temperatures ranging from 25 to 60 °C. After enzymes inactivation (10 min. at 100 °C) and removal of remaining polysaccharides particles, the quantification of reducing sugars was spectrophotometrically performed at A_540_ using bicinchoninic acid (BCA) assay [32]. A calibration curve of d-Glucose (0 to 0.04 g/L) was used as standard. One unit of chitinase activity (U) was considered as the release of 1 µmol of reducing sugar per min.

### 2.4. pH and Temperature Stability

The pH and temperature stability of parental and fusion enzymes were studied according to the same methods used for optimal temperature analysis (see Section 2.3). For temperature stability, enzymes were preliminarily incubated for 30 min at various temperatures ranging from 25 °C to 65 °C. The thermostability was estimated by determining the residual activity obtained for each temperature compared to the higher activity observed at 25 °C. The pH stability for both glucuronan lyase and chitinase activities was evaluated for various pHs ranging from 3 to 8.5. Enzymes were pre-incubated for 30 min in different pH solutions (50 mM potassium acetate buffer for pH between 4 and 7.5, 50 mM MOPS (3-(N-morpholino)propanesulfonic acid) buffer for pH between 6 and 8.5, and 50 mM Tris–HCl between 7 and 8.5), and enzymes activities were measured at 25 °C according to the same methods used for optimal temperature analysis. The higher activity observed for optimal pH was used as reference (100%) to express residual activity at the different pH. 

### 2.5. Kinetic Parameters Analysis of Glucuronan Lyase

Regarding the glucuronan lyase, the kinetic parameters such as the Michaelis constant (Km), the catalytic constant (kcat) and the catalytic efficiency (kcat/Km) were measured at 25 °C, using 0.0625–2 g/L deacetylated glucuronan as substrate in 50 mM potassium acetate buffer (pH 5.5). The Km values were determined using Linewaever-Burk plots. All the assays were repeated in triplicate.

### 2.6. 3D Structure Simulation of Chimeric Enzyme

The prediction of the 3D structures of the parental and fusion enzymes was performed by comparative modeling. The structures of parental enzymes were determined by SWISS Model (https://swissmodel.expasy.org/), the template selected for homology modeling of ThCHIT42 is Endochitinase 42 from *Trichoderma harzarium* ( PDB accession number 6epb.1.A) and the one selected for the modeling of TrGL is Glucuronan Lyase A from *Trichoderma reesei* (PDB accession number 2zzj.1.A). In order to merge the ThCHIT42 and TrGL models of the chimeric proteins, the MultiDomain Assembler (MDA) approach was used by UCFC Chimera software and its computational web services (http://www.rbvi.ucsf.edu/chimera/). The molecular figures were visualized by PyMol (https://pymol.org/) and Ezmol (http://www.sbg.bio.ic.ac.uk/ezmol/). 

## 3. Results

### 3.1. Expression of Fused TrGL-Thchit42 Constructions in Pichia Pastoris

**Figure 2:**
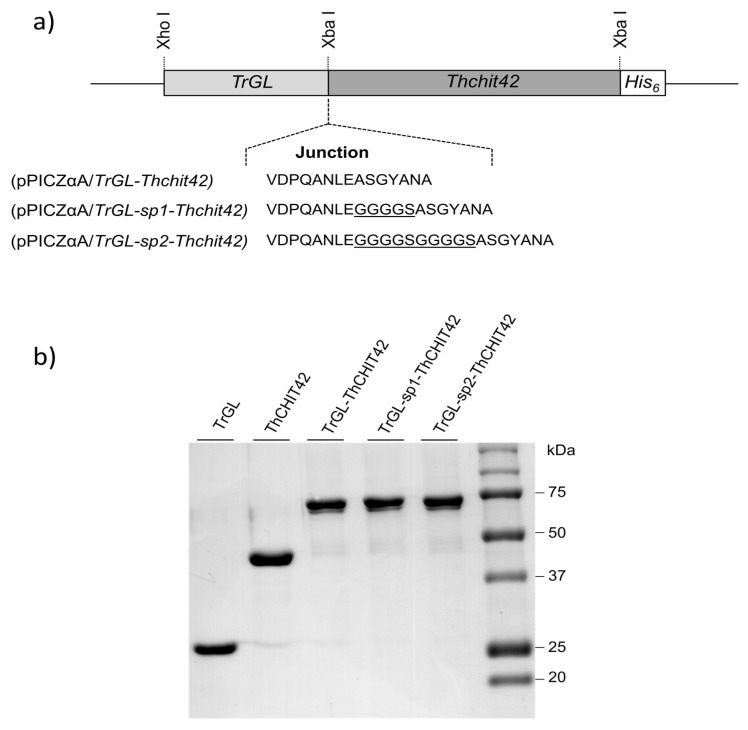
Schematic representation of chimeric constructions with their respective junctions (linkers, underlined) (**a**), and SDS-PAGE gel (12%) analysis of recombinant parental and fused enzymes after purification by Chromatography (IMAC) (**b**).

Three chimeric (TrGL-ThCHIT42, TrGL-sp1-ThCHIT42 and TrGL-sp2-ThCHIT42) and two parental (TrGL and ThCHIT42) constructions (Figure 2a) were carried out in pPICZαA expression vector, and were expressed in *P. pastoris* KM71H. After 72 h methanol induction, extracellular proteins secreted in the supernatant was analyzed by SDS-PAGE and the results showed a single band both for parental and chimeric recombinants (data not shown). The quantification of extracellular protein concentration showed production yields of 96 and 136 mg/mL for, respectively, TrGL and ThCHIT42, and 60, 38 and 40 mg/mL, respectively, for TrGL-Chit42, TrGL-sp1-ThCHIT42 and TrGL-sp2-ThCHIT42. Secreted His-tagged recombinant proteins were purified using Ni-affinity chromatography (IMAC) and analyzed by SDS-PAGE. The result showed a single protein band, slightly lower than 75 kDa for the fusion enzymes which is consistent with the expected size ranging from 72.95 to 73.58 kDa according to the fusion enzymes, and proteins closed to 45 and 30 kDa, respectively, for Histidine-tagged parental proteins ThCHIT42 and TrGL (Figure 2b). 

### 3.2. Enzymatic Activities of TrGL-ThCHIT42 Chimeras

**Figure 3:**
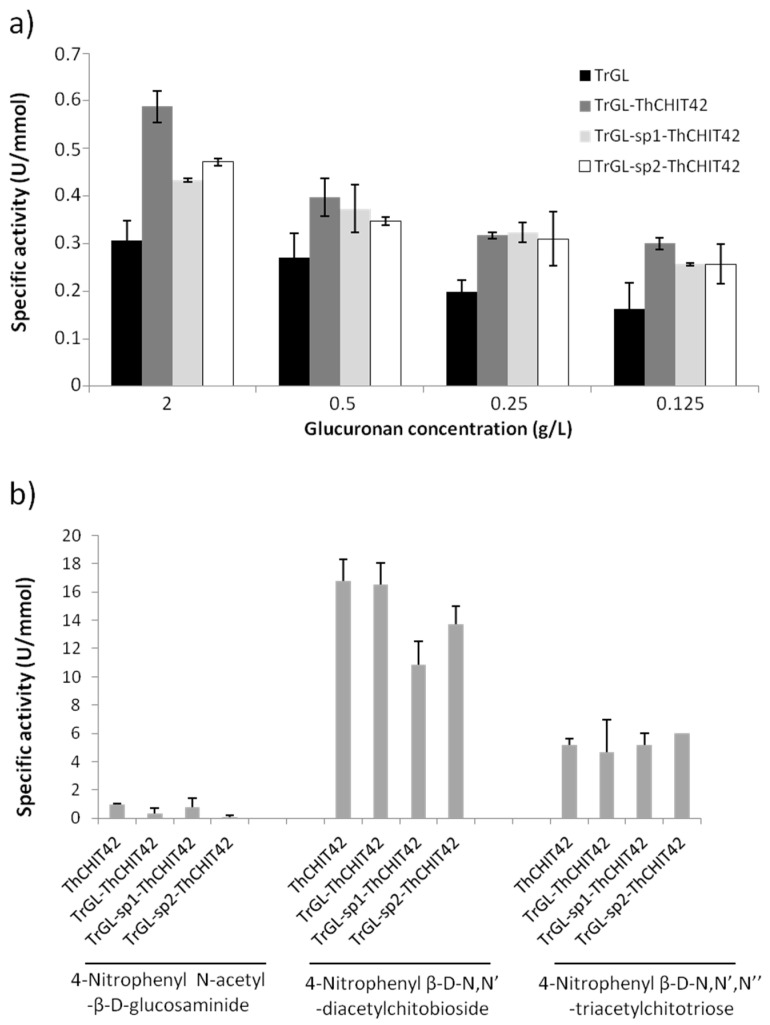
Specific activities for glucuronan lyase obtained for different substrate concentrations (**a**), and for chitinase using three substrates to test endo-type ((β-d-N,N’,N’’-triacetylchitotriose) and exo-type (N-acetyl-β-d-glucosaminidase and N,N’-diacetyl-β-d-chitobioside) chitinase activity (**b**). All the values were issue from three independent experiments (n = 3).

Both glucuronan lyase and chitinase activities were first screened to validate the enzymatic functionality of chimeric enzymes and compared with parental ones. For this purpose, the specific activities were measured and expressed in Units/mmol of enzyme (µmol/min/mmol of enzyme) rather than in U/mg, to respect the equimolarity of enzymatic active sites. Glucuronan lyase activity was determined measuring A_235_ using deacetylated glucuronan (2, 0.5, 0.25 and 0.125 g/L) as substrate. As shown in Figure 3a, glucuronan lyase activity of the three chimeras was not affected by the fusion and appeared more efficient compared to parental TrGL. A substantial increase of specific activities was effectively observed for the different tested substrate concentrations, reaching +46.8% to 91.5% for TrGL-ThCHIT42, +38% to 62.4% for TrGL-sp1-ThCHIT42 and +28.5% to 57% for TrGL-sp2-ThCHIT42. The presence of linkers had no real benefit on enzymatic activity. Considering the chitinolytic activity (Figure 3b), very slight or no N-acetyl-β-d-glucosaminidase activity was detected for both parental and chimeric enzymes, which is consistent with previous studies categorizing ThCHIT42 as an endochitinase [31]. The results obtained for both ThCHIT42 and chimeric enzymes with N,N’-diacetyl-β-d-chitobioside substrate suggested a significant chitobiosidase activity ranging from 10.82 to 16.75 U/mmol, and characteristic of exo-type chitinase family. This chitobiosidase activity was slightly altered (20–30%) for TrGL-sp1-ThCHIT42 and TrGL-sp2-ThCHIT42 chimeras. Endo-chitinase activity measured with β-d- N,N’,N’’-triacetylchitotriose substrate was found substantially equivalent, between 4.70 and 6 U/mmol, for parental and chimeric enzymes.

### 3.3. Kinetic Parameters of Glucuronan Lyase

As no improvement of chitinase activity was observed with the fusions, only kinetic parameters of glucuronan lyase were analyzed to understand the factors behind the increase of lyase activity. As shown Table 2, the parental TrGL and TrGL-ThCHIT42 exhibited a similar Km closed to 0.095 g/L while a slight lower value was obtained for the fusions including the linkers. The higher kcat value obtained for the chimeric enzymes resulted in a significant increase of the efficiency of the fusions, for which the kcat/Km were 1.75- and 1.90-fold higher than for the parental TrGL.

### 3.4. Temperature and pH Stability of TrGL-ThCHIT42 Chimeras vs. Parental Enzymes

**Figure 4:**
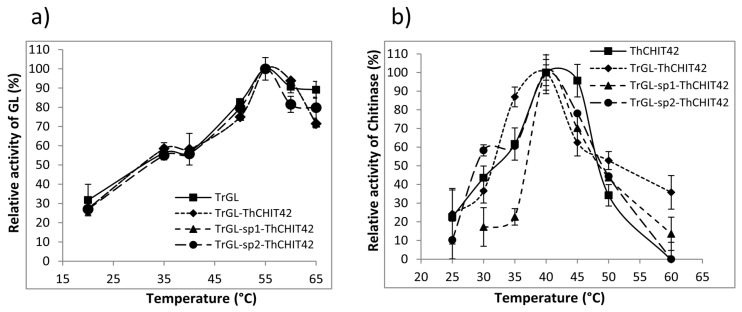
Determination of optimal temperatures of parental and chimeric constructions for glucuronan lyase using deacetylated glucuronan as substrate (**a**) and chitinase (**b**) using colloidal chitin. The values are the average of three independent repeated experiments.

In order to estimate the impact of fusion on biochemical properties of glucuronan lyase and chitinase activities, assays were first carried out to define optimal temperatures of enzymes, using glucuronan and colloidal chitin as substrates. A similar profile of glucuronan lyase activity, observed within the temperature range of 25–65 °C for parental TrGL and fusion enzymes (Figure 4a), confirmed that fusion did not affect GL activity, preserving the optimal temperature at 55 °C. Similarly, parental and chimeric enzymes displayed an identical optimal temperature of 40 °C for chitinase activity (Figure 4b). However, disparate behaviors were observed between the different fusion constructions, as shown, for example, for TrGL-sp1-ThCHIT42, which have a clearly lower activity (about 20%) at 30–35 °C compared to ThCHIT42 (40–60%), whereas an equivalent or higher activity (up to 90%) was observed for TrGL-ThCHIT42 and TrGL-sp2-ThCHIT42. This divergence of chitinase activity between chimeric enzymes might be attributed to the presence and/or nature of linkers (sp1, sp2), which instead seemed to have no impact for glucuronan lyase activity. 

**Figure 5:**
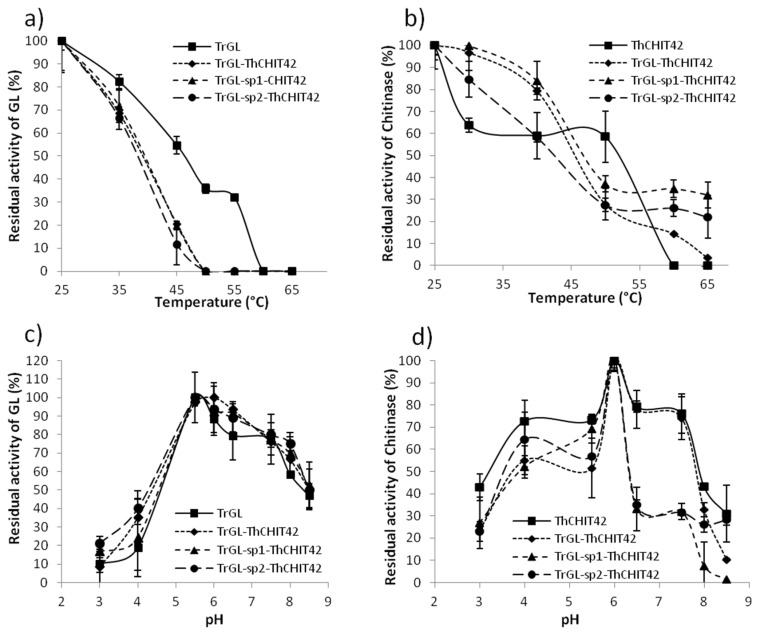
Analysis of thermostability and pH stability of both parental and the fusions. The thermostability of glucuronan lyase (**a**) and chitinase (**b**) was estimated within a range of 25–65 °C, and the residual activity was calculated to the temperature reference (100% at 25 °C). The pH stability was analyzed within the pH range 3–9 for glucuronan lyase (**c**) and chitinase (**d**), and the residual activity was determined using pH optimum (5.5 for glucuronan lyase and 6 for chitinase) as reference (100%). All assays were carried out in triplicate.

The analysis of the enzyme thermostability highlighted an increasing instability, within the overall range of 25–50 °C, of fusion enzyme related to glucuronan lyase activity (Figure 5a). A total loss of activity was demonstrated after an incubation of enzymes during 30 min at 50 °C, whereas about 35% of activity was persistent for parental TrGL. Regarding the chitinase activity, fusion enzymes seemed to be relatively more stable within the range of 25–35 °C than ThCHIT42, for which a significant reduction of about 40% was observed between 25–30 °C, followed by a constant remaining activity (~60%) (Figure 5b). On the other hand, ThCHIT42 maintained 50% of its activity after 30 min at 50 °C, whereas the activity of fusion enzymes dropped to 20–35%. The activity of ThCHIT42 was completely lost at 60 °C, whereas chimeric enzymes, notably TrGL-sp1-ThCHIT42 and TrGL-sp2-ThCHIT42, maintained 20–30% of activity within 60–65 °C.

The optimum pHs of 5.5 and 6, determined, respectively, for TrGL and ThCHIT42, were preserved for overall fusions (Figure 5c,d). Both parental and chimeric enzymes were relatively stable for glucuronan lyase activity for pH ranging from 5 to 8. Considering chitinase activity, fusions enzymes seemed to be more sensitive to pH varying from 3 to 5. The pattern of chitinase activity for pH > 6 was relatively closed between parental ThCHIT42 and TrGL-ThCHIT42, whereas a considerable decline for pH > 6 was observed for fusions TrGL-sp1-ThCHIT42 and TrGL-sp2-ThCHIT42, probably due to the presence of sp1 and sp2 linkers (Figure 5d).

### 3.5. 3D structure of Chimeric Enzymes and Glucuronan Lyase

**Figure 6:**
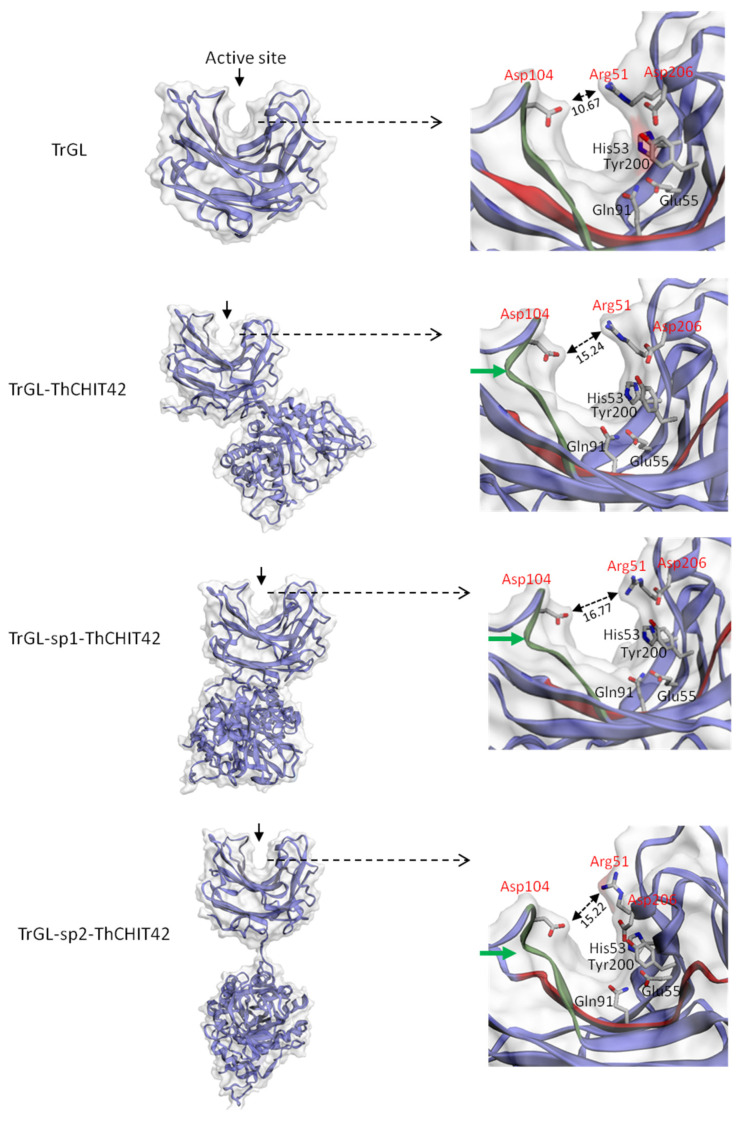
Overall 3D structure simulation of TrGL and chimeric enzymes (left panel) and the conformational structure of the cleft of glucuronan lyase (right panel). The amino acids His53, Glu55, Gln91, Tyr200 and Asp206 are catalytic residues. The residues shown in red are located at the lid of the cleft. The enlargement of the cleft (at the lid level) is indicated by a double arrow (↔), with the distance indicated in Angstrom. A curvature of the chain near the residue Asp104 is shown by a green arrow.

To gain insights into the mechanism of kcat increase observed for glucuronan lyase, simulations of 3D structure was carried for chimeric enzymes and the parental TrGL (Figure 6). The analysis of the conformation at the active site has revealed the conservation between the fusion and parental enzymes of the position of the catalytic residues Arg51, His53, Glu55, Gln91, Tyr200 and Asp206 [33]. However, local conformational differences have been noticed at the cleft, notably a curvature of the chain for fusion enzymes occurring near the residue Asp104. In addition, the catalytic residue Asp 206, located at the lid of the cleft takes orientations different from that of the TrGL (Figure 6). These modifications of Asp206, and potentially the curvature, seem to induce the enlargement of the cleft (between Asp104 and Asp206), which could have a beneficial impact on the accessibility of the substrate. 

## 4. Discussion

Since the last decades, designing and constructing of chimeric enzymes, generally based on the association of two enzymes in a bifunctional protein complex, were widely explored and pursued for different purposes including the research of synergistic or cooperative effects between partners [34,35], the improvement of inherent biochemical properties such as enzymes stability (pH and thermostability) and activity [36,37,38], and the expansion of the specificity to additional substrates [39,40]. In these perspectives, many efforts have been made in the development of fusion enzymes by genetic engineering to optimize the degradation of plants cell wall polymers (polysaccharides, lignin, hemicelluloses, etc.) in plants biomass conversion processes and valorization applications [35,41,42].

In this study, we focused on the analysis of the functionality of different chimeric constructions, combining the fusion of a glucuronan lyase and a chitinase acting independently on two structural polymers of cell-wall of Mucorales fungi, and the determination of the benefits related to biochemical and catalytic properties, gained by the fusion. The results highlighted that both chitinase and glucuronan lyase activities were retained for all chimeric enzymes, without diminution of specific activities compared to parental enzymes. The enzymatic activities in fusion systems can be negatively impacted by different factors such as domain interaction between the partners or the proximity of active sites, which can hamper the substrate accessibility. As showed by the 3D-strucutre modeling (Supplementary Data, S1) of the three chimeric enzymes, the localization at opposite sides of glucuronan lyase and chitinase active sites allowed maintaining intrinsic catalytic activities without apparent conformational constraints. Few studies related to the fusion of a chitinase with another enzyme have been reported. Most of works focused on the fusions with carbohydrate-binding domain (CBD) to improve chitinase performances. These CBD-chitinase constructions were reported unstable [21] or showed a decrease or increase of activity on soluble chitin, according to the nature of the CBD [43,44]. 

The insertion of peptide linkers between the two partners of the fusion is often reported to promote the correct folding of both moieties [45], although the usefulness and the impact of linkers on conformational structure is dependent upon the nature and structure of the fused proteins. No difference on enzyme velocities was observed between the fusions including or not the linkers ((GGGGS)_1_ or (GGGGS)_2_), maybe due to the localization of the catalytic sites at opposite sides. In addition, structural interactions between sp1 and sp2 linkers with ThCHIT42 observed with 3D structure simulations for TrGL-sp1-ThCHIT42 and TrGL-sp2-ThCHIT42, seem to have no impact on enzyme velocities, but may affect other physical properties of the fusion (e.g., stability). Furthermore, the results have shown that the native ThCHIT42 considered as an endochitinase displayed a chitobiosidase activity, slightly lower (20–30%) in chimeric enzymes. This observation was consistent with previous studies [31] and suggested that fusions conserved both endochitinase and exochitinase (chitobiosidase, E.C. 3.2.1.29) activities. 

A significant increase of the glucuronan lyase hydrolytic velocity, ranging between 50% to 90% (according to the substrate concentration), was noticed for the fusions compared to parental enzyme, while hydrolytic velocities of chimeric and parental enzymes were overall similar for the chitinase one. The catalytic efficiency (kcat/Km) of the chimeric glucuronan lyase velocities were about 80% higher than the parental. As Km of chimeric and TrGL enzymes were approximately similar, the increase of velocities would not involve a higher substrate affinity. This gain of activity for chimeric fusion has been previously described in literature such as for chimeric laccase/β-(1,3)(1,4)-glucanase enzyme, and might be attributed to conformational changes leading to improve accessibility to the substrate [34]. The comparative analysis of 3D structure modeling of TrGL and chimeric enzymes has revealed a possible enlargement at the glucuronan active site for the fusion enzymes, induced by the differential orientations of the residue Arg51 located at the lid of the cleft and a curvature close to Asp104. This enlargement at the cleft may contribute to increase the velocity of the chimeric enzymes by increasing the accessibility of the substrate or the release of products.

Considering chitinase, kinetic parameters were not analyzed since any improvement of specific activities was observed for chimeric enzymes. However, as the gain of benefits in fusion systems is dependent on the relative position (N terminus or C terminus) of each partner, it will be relevant to analyze the ThCHIT42-TrGL constructions.

The interest of chimeric construction mainly lies in the acquisition of a better stability to temperature or pH variations [35,46]. In this study, both parental and chimeric enzyme displayed a similar optimal temperature for chitinase and glucuronan lyases, evaluated at 55 °C for glucuronan lyase as previously described [23], and 40 °C for chitinase, consistent with the value obtained for endogen CHIT42 purified from *T. harzanium* [20] and recombinant ThCHIT42 expressed in *P. pastoris* [31]. The thermostability was affected by the fusion, notably for glucuronan lyase activity with a complete loss of activity at 50 °C whereas the parental enzyme retained 35% of its activity, as also previously observed by Konno et al. [24]. The thermostability of chitinase was differentially affected in the chimeric enzymes, which may be correlated to the differential interaction of sp1 and sp2 linkers with the chitinase domains, observed with 3D structure models (Supplementary Data, S1). The chimeric enzymes were more stable than ThCHIT42 at temperature within the range of 25–35 °C and 60–65 °C. Whereas ThCHIT42 maintained 50% of its activity at 50 °C, as previously reported for native chitinase [20], only 20–35% of remaining activity was recorded for the fusion enzymes. The thermostability curve obtained for the parental ThCHIT42 was slightly different with the results from Kidibule et al. [31]. The authors observed a steady decline of ThCHIT42 activity (100% to 0%) between 30–50 °C, while we noted in this study an irregular decline pattern with a total loss of activity at 60 °C. The differences between the experimental procedures could explain this divergence and notably that the ThCHIT42 activity was higher at the temperature of 25 °C used as a reference (100%), compared to the reference temperature (30 °C) employed in the study of Kidibule et al. [31]. The optimum pHs, determined for glucuronan lyase and chitinase (respectively, at 5.5 and 6) were consistent with previous studies [20,23,24,31], and strictly preserved between parental and overall fusion. The pH stability was not affected for glucuronan lyase activity in the chimeric enzymes, whereas their chitinase activities seemed to be more sensitive to pH varying from 3 to 5. At pH > 6, only TrGL-ThCHIT42 conserved an identical profile to parental while the presence of linkers induced a significant loss of activity in TrGL-sp1-ThCHIT42 and TrGL-sp2-ThCHIT42 chimeras. Overall, these results clearly demonstrated that biochemical properties of chitinase were affected by the fusion with glucuronan lyase, which may involve possible inappropriate structural interactions or misfolding due to the proximity of glucuronan lyase or linkers [47]. 

Finally, the construction of a bicatalytic enzyme by the fusion of a glucuronan lyase and a chitinase activity leads to a functional protein complex, with conserved or enhanced catalytic performances. Regarding our results, the fusion TrGL-ThCHIT42 seemed to be the construction offering an interesting compromise between velocity and temperature and pH stability. In perspective, the efficiency of the bifunctional enzyme, notably TrGL-ThCHIT42, on the cell wall degradation of Mucorales should be performed to address a potential application of these chimeras as a biocontrol agent. 

## Figures and Tables

**Table 1:** List of primers used for the fusion constructions.

Gene	Primer	Nucleotide Sequence (5’-3’)	Cloning Sites	Final Plasmid
*TrGL*	XTrGL-Fwd	TTTCTCGAGAAAAGAACCCGCAGCTTCTACAACGACGG	XhoI	pPICZαA/*TrGL*
	XbTrGL-Rev	AAATCTAGATTAAGCCTGGTCAGGGTCGACATCG	XbaI	
*Thchit42*	EThchit42-Fwd	GGGGAATTCGCTAGTGGTTACGCTAACGC	EcorI	pPICZαA/*Thchit42*
	XbThchit42-Rev	AAATCTAGATTAAGACCACTACGAATGTTATC	XbaI	
*Thchit42*	XbThchit42-Fwd	AAATCTAGAGGCTAGTGGTTACGCTAACGCT	XbaI	pPICZαA/*TrGL-Thchit42*
	XbThchit42-Rev	AAATCTAGATTAAGACCACTACGAATGTTATC	XbaI	
*Thchit42*	Xbsp1Thchit42-Fwd	AAATCTAGAG**GGTGGCGGTGGCTCG**GCTAGTGGTTACGCTAACGCT	XbaI	pPICZαA/*TrGL-sp1-Thchit42*
	XbThchit42-Rev	AAATCTAGATTAAGACCACTACGAATGTTATC	XbaI	
*Thchit42*	Xbsp2Thchit42-Fwd	AAATCTAGAG**GGCGGTGGTGGGTCGGGTGGCGGTGGCTCG**GCTAGTGGTTACGCTAACGCT	XbaI	pPICZαA/*TrGL-sp2-Thchit42*
	XbThchit42-Rev	AAATCTAGATTAAGACCACTACGAATGTTATC	XbaI	

Restriction sites are underlined. Sequence coding for linkers sp1 (GGGGS)_1_ and sp2 (GGGGS)_2_ are shown in bold.

**Table 2:** Kinetic parameters of parental and chimeric enzymes for the glucuronan lyase activity. The parameters were calculated from three repeated experiments (n = 3).

Enzymes	kcat [Min^−1^]	Km (g/L)	kcat/Km [Min^−1^. (g/L)^−1^]
TrGL	0.299 ± 0.033	0.096 ± 0.029	3.105
TrGL-ThCHIT42	0.503 ± 0.019	0.092 ± 0.002	5.423
TrGL-sp1-ThCHIT42	0.444 ± 0.014	0.076 ± 0.004	5.847
TrGL-sp2-ThCHIT42	0.493 ± 0.107	0.088 ± 0.024	5.570

## Supplementary Materials

The following are available online at https://www.mdpi.com/2075-1729/10/10/234/s1, Figure S1: Overall 3D structure simulation of parental and chimeric enzymes.
