# Supplementary material for: Biochemical Characterization of a Bifunctional Enzyme Constructed by the Fusion of a Glucuronan Lyase and a Chitinase from Trichoderma sp
Source: Life (Basel). 2020 Oct 8;10(10):234. doi: 10.3390/life10100234 (PMC7601620; doi:10.3390/life10100234)
Supplement: Supplementary file 1 [file life-10-00234-s001.pdf]

life

-Supplementary data S1:

The prediction of the 3D structures of the parental and fusion enzymes was performed by comparative modeling. The structures of parental enzymes were determined by SWISS Model (https://swissmodel.expasy.org/), using Endochitinase 42 from *Trichoderma harzarium* (PDB accession number 6epb.1.A) and Glucuronan Lyase A from *Trichoderma reesei* (PDB accession number 2zzj.1.A) as templates for homology modeling. The merge of the ThCHIT42 and TrGL models of the chimeric proteins was carried out using the MultiDomain Assembler (MDA) (UCFC Chimera software and its computational web services (http://www.rbvi.ucsf.edu/chimera/)). The molecular figures were visualized using PyMol (https://pymol.org/). The results of simulations are shown figure S1. The respective catalytic sites of TrGL and ThCHIT42 are located in opposite sides on the chimeric enzymes (figure S1 b, c and d), which suggested no potential impact on catalytic reactions, as demonstrated by the conservation of functional activities for the chimeric enzymes compared to parental enzymes. While no interaction between TrGL and ThCHIT42 within TrGL-ThCHIT42 was observed (figure S1b), the linkers sp1 and sp2 led to structural interactions with ThCHIT42 regarding TrGL-sp1-ThCHIT42 and TrGL-sp2-ThCHIT42 (figure S1 c and d). These structural interactions could affect physical properties of the chimeric enzymes, and might be a factor behind the decrease of stability (e.g. to temperature and pH), observed for the fusions compared to parental enzymes.

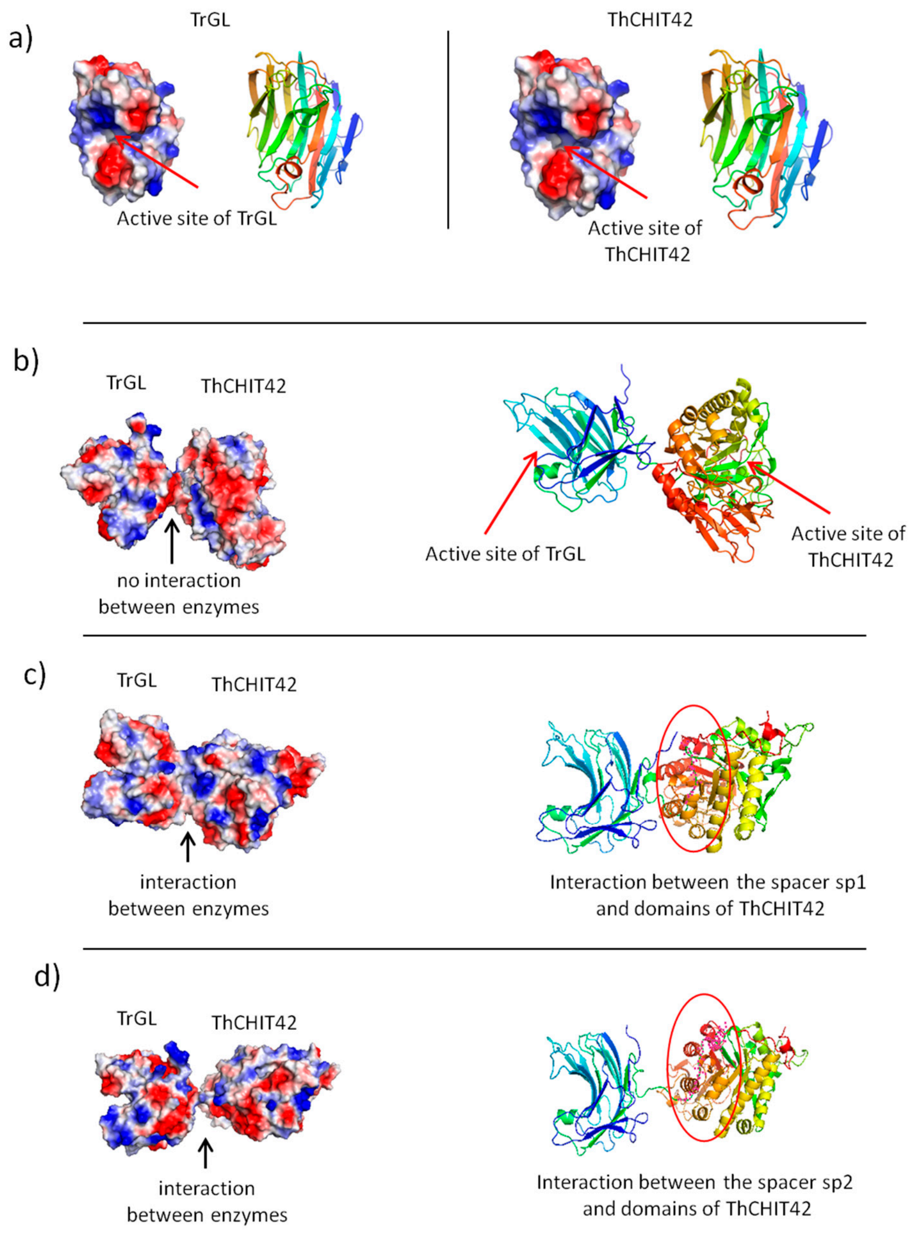

Figure S1. Overall 3D structure simulation of TrGL and ThCHIT42 **(a)** and chimeric enzymes chimeric enzymes TrGL-ThCHIT42 **(b)**, TrGL-sp1-ThCHIT42 **(c)** and TrGL-sp2-ThCHIT42 **(d)**. The prediction of the 3D structures of the parental and fusion enzymes was performed by comparative modeling. The structures of parental enzymes were determined by SWISS Model (https://swissmodel.expasy.org/), using Endochitinase 42 from *Trichoderma harzarium* (PDB accession number 6epb.1.A) and Glucuronan Lyase A from *Trichoderma reesei* (PDB accession number 2zzj.1.A) as templates for homology modeling. The merge of the ThCHIT42 and TrGL models of the chimeric proteins was carried out using the MultiDomain Assembler (MDA) (UCFC Chimera software and its computational web services (http://www.rbvi.ucsf.edu/chimera/)). The molecular figures were visualized using PyMol (https://pymol.org/).

© 2020 by the authors. Submitted for possible open access publication under the terms and conditions of the Creative Commons Attribution (CC BY) license (http://creativecommons.org/licenses/by/4.0/).
